# Cardiovascular and respiratory evaluation in adenosine A_2A_ receptor knockout mice submitted to short‐term sustained hypoxia

**DOI:** 10.1113/EP091221

**Published:** 2023-08-26

**Authors:** Juliana R. Souza, Benedito H. Machado

**Affiliations:** ^1^ Department of Physiology, School of Medicine of Ribeirão Preto University of São Paulo Ribeirão Preto SP Brazil

**Keywords:** A_2A_ receptors, adenosine, cardiovascular and respiratory system, knockout mice, sustained hypoxia

## Abstract

Sustained hypoxia (SH) in mice induces changes in the respiratory pattern and increase in the parasympathetic tone to the heart. Among adenosine G‐protein‐coupled receptors (GPCRs), the A_2A_ receptors are especially important in mediating adenosine actions during hypoxia due to their expression in neurons involved with the generation and modulation of the autonomic and respiratory functions. Herein, we performed an in vivo evaluation of the baseline cardiovascular and respiratory parameters and their changes in response to SH in knockout mice for A_2A_ receptors (A_2A_KO). SH produced similar and significant reductions in mean arterial pressure and heart rate in both wild‐type (WT) and A_2A_KO mice when compared to their respective normoxic controls. Mice from WT and A_2A_KO groups submitted to normoxia or SH presented similar cardiovascular responses to peripheral chemoreflex activation (KCN). Under normoxic conditions A_2A_KO mice presented a respiratory frequency (*f*_R_) significantly higher in relation to the WT group, which was reduced in response to SH. These data show that the lack of adenosine A_2A_ receptors in mice does not affect the cardiovascular parameters and the autonomic responses to chemoreflex activation in control (normoxia) and SH mice. We conclude that the A_2A_ receptors play a major role in the control of respiratory frequency and in the tachypnoeic response to SH in mice.

## Abstract (highlights)

- **What is the central question of this study?**
  Are cardiovascular and respiratory parameters and their changes in response to sustained hypoxia (SH) altered in adenosine A_2A_ receptor knockout mice?
- **What is the main finding and its importance?**
  Cardiovascular parameters and their changes in response to SH were not altered in A_2A_KO mice. The respiratory frequency in A_2A_KO was higher than in WT mice. In response to SH the respiratory frequency increased in WT, while it was reduced in A_2A_KO mice. A_2A_ receptors play a major role in the modulation of respiratory frequency and in the tachypnoeic response to SH in mice.

## 1 INTRODUCTION

Autonomic and respiratory functions are controlled by specific neural networks in order to maintain homeostasis, as well as promote the required neurovegetative adjustments in response to metabolic challenges such as hypoxia (Abboud et al., 1976; Costa et al., 2014; Moreira et al., 2011; Zoccal et al., 2009). Reduction in oxygen supply below the metabolic demand is characterized as hypoxia and represents one of the most challenging conditions for mammals (Costa et al., 2014). The peripheral chemoreceptors located in the carotid bodies (CB) comprise the main sensory system activated in the case of hypoxia, triggering autonomic and respiratory responses in order to keep the $P_{aO_{2}}$ within the physiological range (Barros et al., 2002; Biscoe & Duchen, 1990; Machado, 2001).

Exposure to sustained hypoxia (SH) for hours or days is experienced by individuals ascending to high altitudes, and under this condition important adaptative responses are observed in the cardiovascular and respiratory functions (Calbet, 2003; Hansen & Sander, 2003; Powell, 2007; Powell et al., 1998). Recent studies from our laboratory documented that SH ($F_{iO_{2}}$ 0.1 for 24 h) in mice induced an increase in respiratory activity associated with an augmented parasympathetic tone to the heart (Machado, 2023; Rodrigues et al., 2021; Souza et al., 2022). However, the underlying mechanisms contributing to these changes observed in mice submitted to SH have not yet been evaluated.

Adenosine is an active player in the central nervous system during hypoxic challenges. Under physiological conditions the extracellular levels of adenosine in the brain are relatively low and varied in the range 20–300 nM. However, under challenging conditions such as intense physical activity or hypoxia, the extracellular concentration of adenosine increases into the micromolar range (Borea et al., 2018; Dale et al., 2000; Frenguelli et al., 2003; Lee & Venton, 2018; Liu et al., 2019; Nguyen et al., 2014). Adenosine is also considered one of the most important neuromodulators of synaptic transmission in the brain (Burnstock, 2017; Choudhury et al., 2019; Cunha, 2001; Snyder, 1985). It is also important to note that an increase in the extracellular level of adenosine has been observed after systemic hypoxia in the nucleus tractus solitarii (NTS), the main synaptic station for processing the peripheral chemoreceptor afferents, as well as within regions containing neurons of the ventral respiratory group that are recruited during hypoxic challenges (Barraco et al., 1991; Gourine et al., 2002; Richter et al., 1999; Winn et al., 1981; Yan et al., 1995). Furthermore, it was described that hypoxia induces adenosine release by the CB of rats, which in turn stimulates the carotid chemoreceptors afferents (Conde & Monteiro, 2004; Drumm et al., 2004), representing another important physiological role for adenosine in mediating the autonomic and respiratory responses to chemoreflex activation.

A_2A_ receptors play a key role for adenosine actions during hypoxia for the following reasons: (1) A_2A_ receptors are expressed in neurons and astrocytes in the NTS (Minic et al., 2018; Pickel et al., 2006) and in neurons of the ventral respiratory group (Jiang et al., 2011; Malik et al., 2019; Zaidi et al., 2006), (2) adenosine activates carotid body chemoreceptors via A_2A_ receptors (Fitzgerald et al., 2009; McQueen & Ribeiro, 1986), (3) the antagonism of A_2A_ receptors abolished hypoxia‐induced bradycardia and hypertension in fetal sheep (Koos & Maeda, 2001), and (4) the A_2A_ receptors are involved in the hypoxia‐induced respiratory responses in sheep and lambs (Koos et al., 2005, 2002). However, there is no evidence about the involvement of A_2A_ receptors in the cardiovascular and respiratory adjustments in response to short‐term sustained hypoxia or to peripheral chemoreflex activation in conscious freely moving mice.

Taking into consideration the relevance of adenosine as a signaling molecule during hypoxic challenges and the evidence of an important role of A_2A_ receptors in the cardiovascular and respiratory neural networks under hypoxia, the aims of this study were to evaluate whether the lack of A_2A_ receptors in knockout mice submitted to SH affects (1) the changes in the baseline cardiovascular and respiratory parameters, and (2) the cardiovascular and respiratory responses to chemoreflex activation. To reach these goals, we used control (wild‐type) and adenosine A_2A_ receptor knockout mice sujected to SH, and cardiovascular and respiratory recordings were performed in the conscious freely moving condition.

## 2 METHODS

### 2.1 Ethical approval

All experimental protocols used in this study were approved by the Institutional Ethics Committee on Animal Experimentation of the School of Medicine of Ribeirão Preto, USP (CEUA no. 029/2021). The experimental protocols are also in accordance with the animal ethics principles and regulations of *Experimental Physiology* (Grundy, 2015).

### 2.2 Animals

In this study 34 male adenosine A_2A_ receptor knockout mice (C;129S‐Adora2a^tm1jfc^/J lineage – A_2A_KO, 6–8 weeks, 19–25 g) and 32 male Balb/c mice (6–8 weeks, 19–25 g) provided by Animal Care Facility of the University of São Paulo (USP), campus of Ribeirão Preto, were used. The A_2A_KO mice lineage was originally purchased from The Jackson Laboratory (JAX stock no. 010685; Bar Harbor, ME, USA), and considering that they were bred in a wild‐type (WT) Balb/c mice lineage background (6–8 weeks, ∼20 g), we used this lineage as the genetic background of the A_2A_KO mice. This Balb/c mice lineage was also originally purchased from The Jackson Laboratory (JAX stock no. 000651).

The animals were divided into four groups: (1) Balb/c WT mice under normoxic conditions (Balb/c WT control), (2) Balb/c WT mice submitted to SH (Balb/c WT SH), (3) A_2A_KO mice under normoxic conditions (A_2A_KO control) and (4) A_2A_KO mice submitted to SH (A_2A_KO SH). Before the experiments mice were maintained under standard environmental conditions (23 ± 1°C, 12 h−12 h light–dark cycle) with food and water access ad libitum.

### 2.3 Genotyping of C;129S‐Adora2a^tm1jfc^/J mice

Among the total of 34 knockouts used in the present study, 19 A_2A_KO mice from different litters were randomly selected, and the genotypes were evaluated by PCR analysis from the genomic DNA. Tissue samples were collected from the tails of WT and A_2A_KO mice and the extraction of genomic DNA was performed by incubating the samples in a mix containing 50 μl of extraction buffer + 12.5 μl of preparation buffer (Redextract‐N‐AMP for tissue, XNAT‐1KT, Sigma‐Aldrich, St Louis, MO, USA) at 59°C for 5 min. The following primers were used to amplify the sequences of interest: Common forward: 5ʹ‐GGA CTC CTC GGT GTA CAT‐3ʹ (Thermo Fisher Scientific, Waltham, MA, USA); WT reverse: 5ʹ‐CCC ACA GAT CTA GCC TTA‐3ʹ (Thermo Fisher Scientific); and A2AKO reverse: 5ʹ‐CAT TTG TCA CGT CCT GCA CGA C‐3ʹ (Thermo Fisher Scientific). For each reaction, there was prepared a mix containing 0.6 μl of each primer (10 μM) + 1.2 μl of autoclaved Mili‐Q water + 5 μl of Redextract‐N‐AMP PCR mix (Sigma‐Aldrich). In the sequence, 2 μl of the solution containing the respective DNA was added to each of the reactions. In the negative control, 2 μl of autoclaved Mili‐Q water was added. After running the reactions in a thermocycler (Veriti Dx 96‐well Thermal Cycler, Thermo Fisher Scientific), the PCR products were analysed by 1.5% agarose gel electrophoresis stained with Sybr Safe (Thermo Fisher Scientific).

### 2.4 Arterial and venous catheterization

**FIGURE 1:**
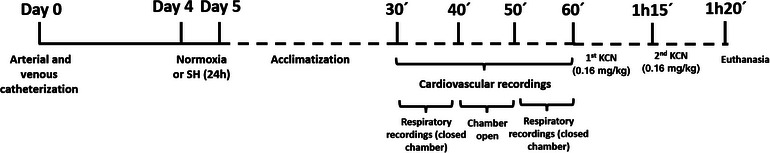
Schematic representation of protocol for cardiovascular and respiratory recordings in conscious freely moving mice submitted to SH or normoxia.

The surgery for implantation of catheters into femoral artery and jugular vein was performed as previously described by Rodrigues, Souza et al. (2021). Under anaesthesia with isoflurane (Isoforine®, Cristália Produtos Químicos Farmacêuticos Ltda., Itapira, SP, Brazil), at a rate of 5% for induction and 1–2% for maintenance, a saline‐filled catheter (MRE‐025, Braintree Scientific, Braintree, MA, USA) was inserted into the femoral artery for measurement of pulsatile arterial pressure (PAP). A polyethylene saline‐filled catheter (LDPE‐PE/05, Scientific Commodities, Lake Havasu City, AZ, USA) was inserted into the jugular vein for KCN injection (0.16 mg/kg; Merck, Darmstadt, Germany). During this surgery, tail pinching and the absence of reflex responses was used to monitor the level of anaesthesia. Both catheters were exteriorized through the back of the animal's scapular waist. After surgery, an antibiotic (Pentabiotic; Fort Dodge Saúde Animal Ltda., Campinas, SP, Brazil) was administered (0.2 ml of 1.2 million IU, i.m.). Mice were maintained under observation by the investigator for at least 2 h, and then were housed in individual cages for 4 days to recover from the anaesthetic and surgical stresses (Figure 1).

### 2.5 Sustained hypoxia

On the fourth day after the surgery for arterial and venous catheterization, A_2A_KO and WT mice were submitted to SH or a normoxic protocol (Figure 1). Mice from the SH group kept in individual cages were placed inside polymethylmethacrylate (Plexiglas®) chambers (volume = 210 litres) equipped with oxygen (O_2_) and nitrogen (N_2_) injectors and sensors of the fraction of inspired O_2_ ($F_{iO_{2}}$). For SH mice $F_{iO_{2}}$ was maintained at 0.1 for 24 h by a computerized system (Oxycycler, Biospherix, Redfield, NY, USA) controlling the injection of O_2_ or N_2_ (Maxiair, Ribeirão Preto, SP, Brazil) inside the chambers via solenoid valves (Oxycycler (Model A84XOV) Biospherix), which were automatically operated by installed software (AnaWin 2, version 2.4.17). A_2A_KO and WT mice from control groups were maintained inside a similar chamber under normoxia ($F_{iO_{2}}$ = 0.208) for 24 h.

### 2.6 Cardiovascular and respiratory recordings in conscious freely moving mice

At the end of the SH or normoxic protocols, the arterial catheter was connected to a pressure transducer (MLT0380; ADInstruments, Bella Vista, NSW, Australia) attached to an amplifier (Bridge Amp, ML221; ADInstruments). Pulsatile arterial pressure (PAP), mean arterial pressure (MAP) and heart rate (HR) signals were acquired by a computerized system (PowerLab 4/25 ML845; ADInstruments) and recorded on a computer (sampling rate: 1 kHz) using an acquisition software (LabChart 5, ADInstruments). Baseline cardiovascular parameters were recorded for 60 min in room air, but the first half hour of recordings was not considered in the data analyses due to possible stress of the animals in response to the manipulation for connecting the catheteres (Figure 1).

The respiratory parameters were evaluated using a whole‐body plethysmography approach (Malan, 1973) in parallel to the baseline cardiovascular recordings. Under these conditions, for each mouse placed inside a sealed acrylic plethysmographic chamber (1 litre), the respiratory‐related oscillations in the pressure inside the chamber were detected by a high‐sensitivity differential pressure transducer (ML141 spirometer, ADInstruments). The signals were processed by a data acquisition system (PowerLab 4/25 ML845; ADInstruments) and recorded on a computer (sampling rate: 1 kHz) via LabChart software (v.5; ADInstruments). The respiratory volume calibration was performed using a syringe to inject 1 ml of air inside the chamber. Temperatures inside and outside the chamber were continuously monitored. After the 30 min of adaptation to the environment by the mice, the chamber was closed and the respiratory variables were recorded in two series of 10 min each, interspersed for periods of 10 min in which the chamber was opened to avoid a major increase of CO_2_ (Figure 1). Tidal volume (*V*_T_) and respiratory frequency (*f*_R_) were calculated as described by Malan (1973), and ventilation (${\dot{V}}_{E}$) was obtained offline as the product of *V*_T_ and *f*_R_. The parameters were analysed using periods of respiratory recordings in which mice were quiet and not exploring the cage.

### 2.7 Activation of peripheral chemoreceptors in conscious freely moving mice

After 60 min of baseline cardiovascular and respiratory recordings, potassium cyanide (KCN, 0.16 mg/kg) was injected (i.v.) to activate peripheral chemoreflex, as described by Franchini & Krieger (1993) KCN was injected twice with a 15‐min time interval between injections (Figure 1). The maximum changes in HR and MAP were quantified as an average of the responses to two activations and the data between groups were compared. At the end of recordings, mice were killed using an injection of a high concentration of the anaesthetic urethane (Sigma‐Aldrich, 2 g kg^−1^, i.v.) (Figure 1).

### 2.8 Arterial blood gases and biochemical parameters analysis in conscious freely moving mice

**FIGURE 2:**
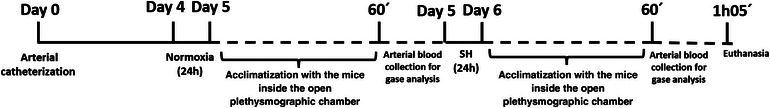
Schematic representation of protocol for arterial blood collections before and after SH in conscious freely moving mice.

In distinct groups of A_2A_KO and WT mice, with the femoral artery previously catheterized, a sample of arterial blood (∼90 μl) was collected via the arterial catheter before and after exposure to SH for arterial blood gas and biochemical parameter analysis (Figure 2). Using the i‐STAT CG4+ gasometry cartridge (REF 03P85‐25) and its i‐STAT analyser (Abbott, Chicago, IL, USA), we measured pH, partial pressure of oxygen ($P_{O_{2}}$), partial pressure of carbon dioxide ($P_{CO_{2}}$), oxygen saturation index ($S_{O_{2}}$) and concentration of bicarbonate (HCO_3_^−^) present in arterial blood. After the initial arterial blood gas analysis (before SH exposure), mice were submitted to the SH protocol, and at the end of the SH protocol a new sample of arterial blood was collected for arterial blood gas analysis as described above. The blood sample was collected before and after SH using a syringe (1 ml) attached to the arterial catheter, in which a small negative pressure was carefully applied to collect 90 μl of arterial blood. This procedure was performed in a room air environment with the mice inside the open plethysmographic chamber and after a period of acclimatization of the animals (∼1 h) to avoid any additional stress to the animal (Figure 2). At the end of the blood sample collections, mice were killed using an injection of a high concentration of the anaesthetic urethane (2 g kg^−1^, i.a.; Sigma‐Aldrich; Figure 2).

### 2.9 Statistical analysis

Data are expressed as means ± standard deviation (SD). The data were analysed using two‐way analysis of variance (two‐way ANOVA). Repeated‐measures analysis of arterial blood gases and biochemical parameters data were performed by fitting a mixed‐effects model. The two‐way ANOVA results for the two individual factors (defined as ‘mice’ to determine the main effects of the absence of A_2A_ receptors, and defined as ‘SH’ to determine the main effects of SH exposure) and interaction (mice vs. SH) are reported. Bonferroni's post‐hoc comparison test was used to report the differences among groups. Differences were considered statistically significant when *P* ≤ 0.05. All graphical and statistical analysis was performed using GraphPad Prism program (version 8, GraphPad Software, La Jolla, CA, USA).

## 3 RESULTS

### 3.1 Genotyping of C;129S‐Adora2a^tm1jfc^/J mice

**FIGURE 3:**
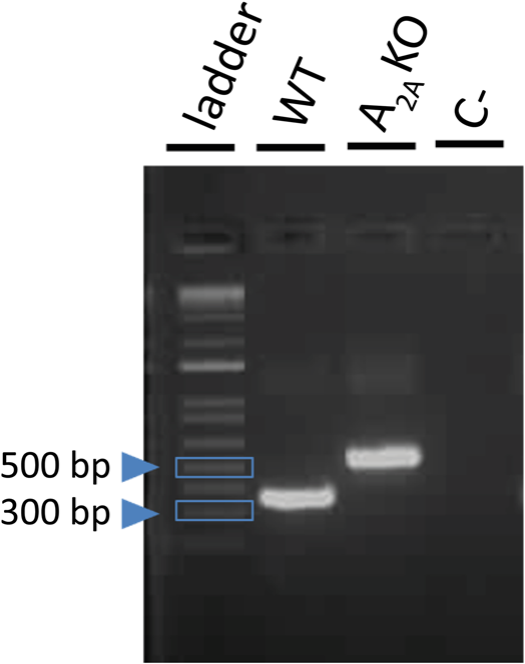
Representative genotype results for A_2A_KO and Balb/c WT mice. DNA ladder and PCR products for one representative wild‐type mouse and one adenosine A_2A_ receptor knockout mouse. C−: negative control. Blue arrows are pointing to 500 and 300 bp weight bands.

Figure 3 shows the genotype of a representative WT control mouse and a representative A_2A_KO mouse. Gel bands of about 550 bases pairs (bp) were observed for knockout mice, gel bands of about 364 bp for wild‐type mice, and no staining in the negative control (C−; no sample). These findings are in agreement with the expected results informed by The Jackson Laboratory from which this lineage was originally purchased (https://www.jax.org/Protocol?stockNumber=010685&protocolID=23548), confirming that the animals used in this study are homozygous knockouts for adenosine A_2A_ receptors subtype.

### 3.2 Cardiovascular parameters

**FIGURE 4:**
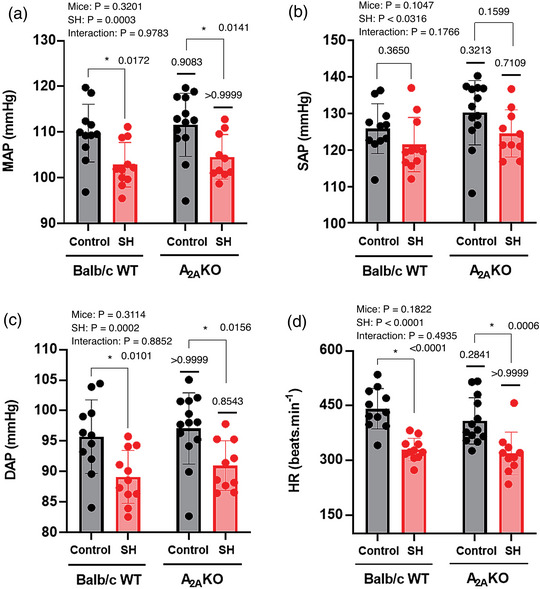
Cardiovascular parameters of conscious freely moving Balb/c WT and A_2A_KO mice submitted to SH or normoxia. Average values of mean arterial pressure (MAP, a), systolic arterial pressure (SAP, b), diastolic arterial pressure (DAP, c) and heart rate (HR, d) of mice from Balb/c WT control group (*n* = 11), Balb/c WT SH group (*n* = 11), A_2A_KO control group (*n* = 13) and A_2A_KO SH group (*n* = 10). Two‐way ANOVA followed by Bonferroni post‐hoc test to compare differences between groups. *P*‐values for individual factors, interaction and post‐hoc comparison test are indicated. *Different from the respective normoxia control group (*P* < 0.05).

MAP, systolic arterial pressure (SAP), diastolic arterial pressure (DAP) and HR were evaluated in Balb/c WT control (*n* = 11) and SH (*n* = 11) mice, and in A_2A_KO control (*n* = 13) and SH (*n* = 10) mice. The cardiovascular parameteres MAP, SAP, DAP and HR were similar in A_2A_KO mice and Balb/c WT mice (Mice effect *P*‐values: 0.3201, 0.1047, 0.3114 and 0.1822), but significantly lower in mice submitted to SH in relation to those maintained under normoxia (SH effect *P*‐values: 0.0003, 0.0316, 0.0002 and <0.0001). No interaction of mice vs. SH was observed (Interaction effect *P*‐values: 0.9787, 0.1766, 0.8852 and 0.4935; Figure 4a–d).

The post‐hoc comparison test shows that WT mice submitted to SH (Balb/c WT SH group) presented a significant reduction in MAP (102 ± 5 vs. 109 ± 6 mmHg, *P* = 0.0172), DAP (89 ± 4 vs. 95 ± 6 mmHg, *P* = 0.0101) and HR (329 ± 31 vs. 441 ± 55 beats min^−1^, *P* < 0.0001), with no significant changes in SAP (121 ± 7 vs. 125 ± 7 mmHg; *P* = 0.3650), when compared to WT mice maintained in normoxia (Balb/c WT control group; Figure 4a–d). Similarly, A_2A_KO mice submitted to SH (A_2A_KO SH group) presented a significant reduction in MAP (104 ± 5 vs. 111 ± 7 mmHg; *P* = 0.0141), DAP (90 ± 4 vs. 97 ± 6 mmHg, *P* = 0.0156) and HR (318 ± 59 vs. 408 ± 64 beats min^−1^, *P* = 0.0006), but no significant changes in SAP (124 ± 6 vs. 130 ± 9 mmHg, *P* = 0.1599) when compared to A_2A_KO mice maintained in normoxia (A_2A_KO control group; Figure 4a–d).

MAP (111 ± 7 vs. 109 ± 6 mmHg, *P* = 0.9083), SAP (130 ± 9 vs. 125 ± 7 mmHg, *P* = 0.3213), DAP (97 ± 6 vs. 95 ± 6 mmHg, *P* > 0.9999) and HR (408 ± 64 vs. 441 ± 55 beats min^−1^, *P* = 0.2841) evaluated under normoxic conditions in WT and A_2A_KO mice were not statistically different. Likewise, the MAP (104 ± 5 vs. 102 ± 5 mmHg, *P* > 0.9999), SAP (124 ± 6 vs. 121 ± 7 mmHg, *P* = 0.7109), DAP (90 ± 4 vs. 89 ± 4 mmHg, *P* > 0.8543) and HR (318 ± 59 vs. 329 ± 31 beats min^−1^, *P* > 0.9999) evaluated after SH in WT and A_2A_KO mice were also not statistically different (Figure 4a–d). Therefore, WT and A_2A_KO mice presented similar reduction in MAP, DAP and HR in response to SH.

### 3.3 Respiratory parameters

**FIGURE 5:**
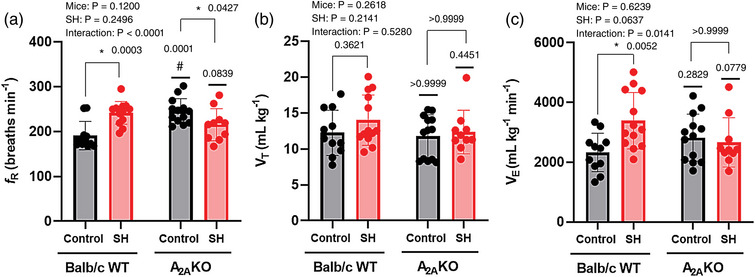
Respiratory parameters of conscious freely moving Balb/c WT and A_2A_KO mice submitted to SH or normoxia (control). Average values of respiratory frequency (*f*_R_, a), tidal volume *(V*_T_, b), and ventilation (${\dot{V}}_{E}$, c) of mice from Balb/c WT control group (*n* = 11), Balb/c WT SH group (*n* = 13), A_2A_KO control group (*n* = 13) and A_2A_KO SH group (*n* = 10). Two‐way ANOVA followed by Bonferroni post‐hoc test to compare differences between groups. *P*‐values for individual factors, interaction and post‐hoc comparison test are indicated. *Different from the respective normoxia control group (*P* < 0.05). #Different from the respective genetic control (*P* < 0.05).

The respiratory parameters *f*_R_, *V*_T_ and ${\dot{V}}_{E}$ were evaluated in Balb/c WT control (*n* = 11) and SH (*n* = 13), and in A_2A_KO control (*n* = 13) and SH (*n* = 10) mice. The parameteres *f*_R_ and ${\dot{V}}_{E}$ in A_2A_KO and Balb/c WT mice were similar (Mice effect *P*‐values: 0.12 and 0.6239), as well as in the groups of mice submitted to SH when compared to those maintained under normoxia (SH effect *P*‐values: 0.2498 and 0.0637). However, the interaction of mice versus SH was different (Interaction *P*‐values: <0.0001 and 0.0141). The parameter *V*_T_ was similar among groups for all factors (Mice effect *P* = 0.2618, SH effect *P* = 0.2141, and Interaction *P* = 0.5280; Figure 5a–c).

The post‐hoc comparison test shows that SH in Balb/c WT mice produced a significant increase in *f*_R_ (241 ± 25 vs. 191 ± 31 breaths min^−1^, *P* = 0.0003) and ${\dot{V}}_{E}$ (3390 ± 944 vs. 2328 ± 641 ml kg^−1^ min^−1^ *P* = 0.0052), with no change in *V*_T_ (14 ± 3 vs. 12 ± 3 ml kg^−1^, *P* = 0.3621) in relation to WT mice maintained in normoxia (Balb/c WT control group; Figure 5a–c). SH in A_2A_KO mice produced a significant decrease in *f*_R_ (215 ± 35 vs. 245 ± 27 breaths min^−1^, *P* = 0.0427) and no changes in *V*_T_ (12 ± 3 vs. 11 ± 3 ml kg^−1^, *P* > 0.9999) and ${\dot{V}}_{E}$ (2663 ± 820 vs. 2819 ± 784 ml kg^−1^ min^−1^, *P* > 0.9999) in relation to A_2A_KO mice maintained in normoxia (A_2A_KO control group; Figure 5a–c).

Interestingly, under normoxia (control) the *f*_R_ (245 ± 27 vs. 191 ± 31 breaths min^−1^, *P* = 0.0001) of A_2A_KO mice was significantly higher than in Balb/c WT mice, while no differences were observed in *V*_T_ (11 ± 3 vs. 12 ± 3 ml kg^−1^, *P* > 0.9999) and ${\dot{V}}_{E}$ (2819 ± 784 vs. 2328 ± 641 ml kg^−1^ min^−1^; *P* = 0.2829). The *f*_R_ (215 ± 35 vs. 241 ± 25 breaths min^−1^, *P* = 0.0839), *V*_T_ (12 ± 3 vs. 14 ± 3 ml kg^−1^, *P* = 0.4451) and ${\dot{V}}_{E}$ (2663 ± 820 vs. 3390 ± 944 ml kg^−1^ min^−1^, *P* = 0.0779) evaluated after SH were similar between A_2A_KO and WT mice (Figure 5a–c). It is important to note that (1) the *f*_R_ of A_2A_KO mice (245 ± 27 breaths min^−1^) was significantly higher than that observed in WT mice (191 ± 31 breaths min^−1^) and similar to that observed in WT mice after SH (241 ± 25 breaths min^−1^), and (2) in mice lacking the adenosine A_2A_ receptors (A_2A_KO) the exposure to SH, in opposition to that observed in WT mice, acctually reduced the *f*_R_ when compared with normoxia (control).

### 3.4 Cardiovascular responses to peripheral chemoreceptors activation

**FIGURE 6:**
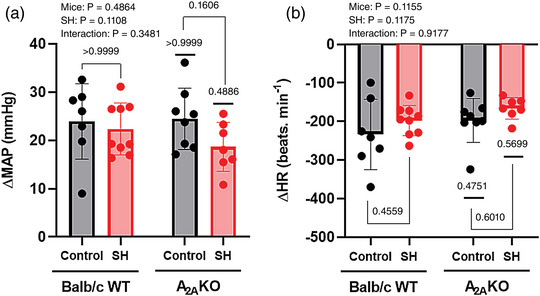
Cardiovascular responses to peripheral chemoreceptor activation with KCN of conscious freely moving Balb/c WT and A_2A_KO mice maintained in normoxia or submitted to SH. Average values of increase in arterial pressure (ΔMAP, a) and heart rate reduction (ΔHR, b) in response to peripheral chemoreceptor activation of mice from Balb/c WT control group (*n* = 7), Balb/c WT SH group (*n* = 9), A_2A_KO control group (*n* = 8) and A_2A_KO SH group (*n* = 7). Two‐way ANOVA followed by Bonferroni post‐hoc test to compare differences between groups. *P*‐values for individual factors, interaction and post‐hoc comparison test are indicated.

The increase in MAP (ΔMAP) and bradycardia (ΔHR) in response to peripheral chemoreflex activation with KCN (0.16 mg kg^−1^, i.v.) were evaluated in Balb/c WT control (*n* = 7) and SH (*n* = 9) mice and in A_2A_KO control (*n* = 8) and SH (*n* = 7) mice. The changes in MAP and HR in A_2A_KO compared to Balb/c WT mice were similar (Mice effect *P*‐values: 0.4864 and 0.1155), as well as in mice submitted to SH in relation to those maintained under normoxia (SH effect *P*‐values: 0.1108 and 0.1175). No difference was observed in the interaction mice versus SH (Interaction *P*‐values: 0.3481 and 0.9177; Figure 6a,b).

The post‐hoc comparison test shows that SH produced no changes in the pressor and bradycardic responses to peripheral chemoreflex activation with KCN in either WT (Balb/c WT SH vs. Balb/c WT control, ΔMAP: 22 ± 5 vs. 23 ± 8 mmHg, *P* > 0.9999, ΔHR: −197 ± 39 vs. −233 ± 91 beats min^−1^, *P* = 0.4559) or A_2A_KO mice (A_2A_KO SH vs. A_2A_KO control, ΔMAP: 18 ± 5 vs. 24 ± 6 mmHg, *P* = 0.1606, ΔHR: −166 ± 28 vs. −197 ± 57 beats min^−1^, *P* = 0.6010, Figure 6a, b). A_2A_KO mice also showed magnitudes of pressor and bradycardic responses to peripheral chemoreflex activation with KCN under normoxia (A_2A_KO control vs. Balb/c WT control, ΔMAP: 24 ± 6 vs. 23 ± 8 mmHg, *P* > 0.9999, ΔHR: −197 ± 57 vs. −233 ± 91 beats min^−1^, *P* = 0.4751) and after SH (A_2A_KO SH vs. Balb/c WT SH, ΔMAP: 18 ± 5 vs. 22 ± 5 mmHg, *P* = 0.4886, ΔHR: −166 ± 28 vs. −197 ± 39 beats min^−1^, *P* = 0.5699) similar to Balb/c WT mice (Figure 6a, b).

### 3.5 Blood gases analysis and biochemical parameters in arterial blood

**FIGURE 7:**
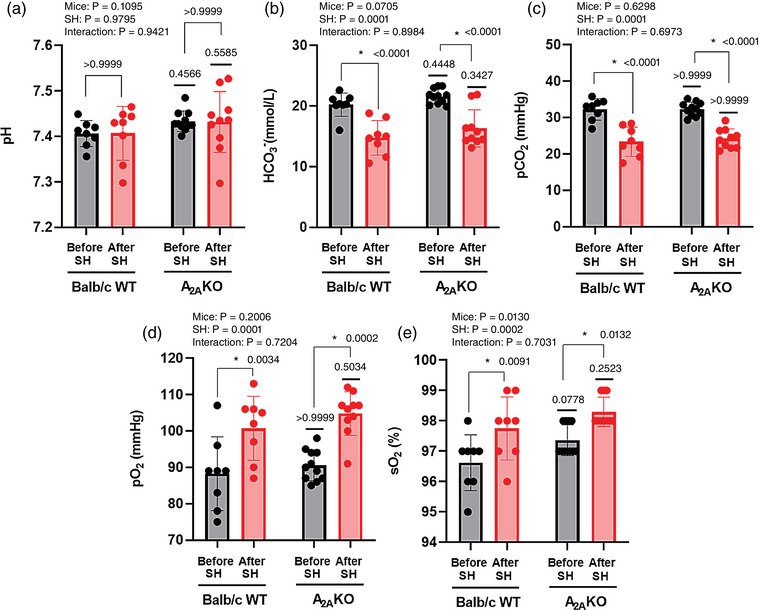
Blood gases and biochemical parameters in arterial blood of conscious freely moving Balb/c WT and A_2A_KO mice before and after SH. Average values of pH (a), bicarbonate concentration (HCO_3_^−^, b), partial pressure of carbon dioxide ($P_{CO_{2}}$, c), partial pressure of oxygen ($P_{O_{2}}$, d), and oxygen saturation ($S_{O_{2}}$, e) in arterial blood of WT mice before (*n* = 8) and after SH (*n* = 8), and A_2A_KO mice before (*n* = 11) and after SH (*n* = 10). Mixed‐effects model analysis for repeated‐measures followed by Bonferroni post‐hoc test to compare differences between groups. *P*‐values for individual factors, interaction and post‐hoc comparison test are indicated. *Different from the measurement before SH (*P* < 0.05).

Arterial blood gases and biochemical parameters were evaluated in WT mice before (*n* = 8) and after SH (*n* = 8), and in A_2A_KO mice before (*n* = 11) and after SH (*n* = 10). The pH was similar for all factors (Mice effect *P* = 0.1095, SH effect *P* = 0.9795, Interaction *P* = 0.9421). The HCO_3_^−^, $P_{CO_{2}}$ and $P_{O_{2}}$ were similar in A_2A_KO mice compared to Balb/c WT mice (Mice effect *P*‐values: 0.0705, 0.6298 and 0.2006), but the HCO_3_^−^ and $P_{CO_{2}}$ were lower in mice after SH in comparison with before SH (SH effect *P*‐values: <0.0001 and <0.0001), while $P_{O_{2}}$ was higher (SH effect *P*‐value < 0.0001). No interaction of mice versus SH was observed for these three parameters (Interaction effect *P*‐values: 0.8984, 0.6973 and 0.7204). The $S_{O_{2}}$ was higher in A_2A_KO mice compared to Balb/c WT mice (Mice effect *P* = 0.0130), as well as in mice after SH in comparison with before SH (SH effect *P* = 0.0002). No interaction of mice versus SH was observed (Interaction effect *P* = 0.7031; Figure 7a–d).

The post‐hoc comparison test shows that SH produced similar effects on arterial gas blood levels and biochemical parameters in both WT and A_2A_KO mice. In Balb/c WT mice, SH produced a significant decrease in $P_{CO_{2}}$ (23 ± 4 vs. 32 ± 3 mmHg, *P* < 0.0001) and HCO_3_^−^ (14 ± 2 vs. 20 ± 2 mmol l^−1^, *P* < 0.0001), an increase in $P_{O_{2}}$ (100 ± 9 vs. 88 ± 10 mmHg, *P* = 0.0034) and $S_{O_{2}}$ (97 ± 1 vs. 96 ± 0.9%, *P* = 0.0091), with no changes in arterial blood pH (7.4 ± 0.06 vs. 7.4 ± 0.03, *P* > 0.9999) when compared to normoxia conditions (Balb/c WT control group). Similarly, in A_2A_KO mice SH produced a significant decrease in $P_{CO_{2}}$ (24 ± 3 vs. 32 ± 2 mmHg; *P* < 0.0001) and HCO_3_^−^ (16 ± 3 vs. 21 ± 1 mmol l^−1^; *P* < 0.0001), an increase in $P_{O_{2}}$ (105 ± 6 vs. 90 ± 4 mmHg, *P* = 0.0002) and $S_{O_{2}}$ (98 ± 0.5 vs. 97 ± 0.5%, *P* = 0.0132), and no changes in arterial blood pH (7.43 ± 0.07 vs. 7.43 ± 0.02 *P* > 0.9999) when compared to normoxia conditions (A_2A_KO control group; Figure 7a–e).

No differences were observed in arterial blood pH (7.43 ± 0.02 vs. 7.4 ± 0.03, *P* = 0.4566), $P_{CO_{2}}$ (32 ± 2 vs. 32 ± 3 mmHg; *P* > 0.9999), HCO_3_^−^ (21 ± 1 vs. 20 ± 2 mmol l^−1^; *P* = 0.4448), $P_{O_{2}}$ (90 ± 4 vs. 88 ± 10 mmHg; *P* > 0.9999) and $S_{O_{2}}$ (97 ± 0.5 vs. 96 ± 0.9%, *P* = 0.0778) between A_2A_KO and Balb/c WT mice under normoxia. Likewise, after SH no differences were observed in arterial blood pH (7.43 ± 0.07 vs. 7.4 ± 0.06, *P* = 0.5585), $P_{CO_{2}}$ (24 ± 3 vs. 23 ± 4 mmHg, *P* > 0.9999), HCO_3_^−^ (16 ± 3 vs. 14 ± 3 mmol l^−1^, *P* = 0.3427), $P_{O_{2}}$ (105 ± 6 vs. 100 ± 9 mmHg; *P* = 0.5034) and $S_{O_{2}}$ (98 ± 0.5 vs. 97 ± 1%, *P* = 0.2523) between A_2A_KO and Balb/c WT mice (Figure 7a–e).

## 4 DISCUSSION

In the present study we used knockout mice for adenosine A_2A_ receptors and Balb/c WT mice as their controls in order to evaluate to what extent the lack of A_2A_ receptors affects (1) the cardiovascular and respiratory parameters, (2) the changes in these parameters in mice submitted to SH and (3) the cardiovascular and respiratory responses to peripheral chemoreflex activation. Our main findings are the following: (1) SH produced similar effects on cardiovascular parameters of both knockout and WT mice, indicating that the A_2A_ receptors play no major role in the cardiovascular changes observed in mice in response to SH, (2) under normoxia A_2A_KO mice presented respiratory frequency significantly higher than in Balb/c WT controls, suggesting that these receptors are important for the generation and/or modulation of respiratory activity, (3) in contrast to the observed increase in WT mice, the exposure to SH reduced the respiratory frequency of A_2A_KO mice, suggesting an important role of these receptors in the tachypnoeic response to SH, and (4) the absence of A_2A_ receptores produced no changes in the magnitudes of pressor and bradycardic responses to peripheral chemoreflex activation in control (normoxia) and SH mice, indicating these receptors are not playing a role in the neurotransmission/neuromodulation of autonomic responses of this reflex.

Although under conscious freely moving condition we observed no differences in the cardiovascular responses to SH and to chemoreflex activation when comparing A_2A_KO and their Balb/c WT control mice, there is evidence in favour of a modulatory role of these receptors in brainstem areas containing neurons directly involved with the generation and modulation of the autonomic activity such as NTS, nucleus ambiguus and rostral ventrolateral medulla (Barraco et al., 1990; Minic et al., 2015, 2018; Phillis et al., 1997; Scislo et al., 2001; Scislo & O'Leary, 2005, 2006; Thomas et al., 2000), as well as the contribution of adenosine and its A_2_ receptors in the signalling transduction that occurs in the CB (Conde et al., 2017; Lahiri et al., 2007; Monteiro & Ribeiro, 1987; Sacramento et al., 2018, 2019).

Considering the cardiovascular parameters under control conditions (normoxia), our findings showed similar levels of MAP, SAP, DAP and HR in WT and A_2A_KO mice. These findings are in accordance with previous studies showing no changes in the baseline cardiovascular parameters in the absence of expression of adenosine A_2A_ receptors in mice (Meriño et al., 2020; Sakata et al., 2005; Sehba et al., 2010). Considering the cardiovascular changes in response to SH, the significant reduction in the HR observed in both A_2A_KO and Balb/c WT mice submitted to SH may indicate an important change in the parasympathetic tone to the heart, since we recently documented that C57BL/c mice submitted to SH presented a similar reduction in HR after SH, which was due to a significant increase in the parasympathetic tone to the heart (Machado, 2023; Rodrigues, Souza et al., 2021; Souza et al., 2022).

With respect to the reduction in MAP in both A_2A_KO and WT mice submitted to SH we suggest that it was mainly due to the reduction in DAP (London & Guerin, 1999; London & Pannier, 2010; Vlachopoulos & O'Rourke (2000). The observed reduction in HR may also have made some contribution to this fall in MAP because it can impact on the cardiac output (CO). However, a study performed by Janssen et al. (2002) demonstrated that in mice the fluctuations in CO are determined more by changes in stroke volume (SV) than by changes in HR levels. The reduction in DAP of mice submitted to SH, in turn, may be the result of an overall vasodilatation rather than a reduction in the sympathetic outflow to the vessels. Adenosine acting on its A_2A_ receptors play an important role in the vascular smooth muscle modulating the vascular tone (Guieu et al., 2020; Iwamoto et al., 1994; Reiss et al., 2019; Shryock & Belardinelli, 1997), which could be linked to local mechanism responsible for the reduction in DAP observed in mice submitted to SH. However, the average values of DAP under normoxia as well as after SH were similar between WT and A_2A_KO mice, suggesting the A_2A_ receptors in the vascular smooth muscle are not involved in the reduction of DAP following SH in WT mice.

In relation to the respiratory parameters, we observed that under normoxia (control) the A_2A_ receptor knockout mice presented a respiratory frequency significantly higher, indicating a key role of these receptors in the maintenance of the basal respiratory activity. We suggest that A_2A_ receptors contribute to enhancing a possible inhibitory input to neurons generating the respiratory rhythm, which apparently is not active in mice lacking these receptors (A_2A_KO). A_2A_ receptors are stimulatory G protein (Gs)‐coupled receptors and their expression in GABAergic neurons contributes to the inhibitory modulation of the neural network (Borea et al., 2018; Corsi et al., 1999; Cunha & Ribeiro, 2000; Ochi et al., 2000). Our suggestion is based upon previous pharmacological studies performed in rats and pigs showing that microinjection of the A_2A_ receptors agonist (CGS‐21680) into the 4th ventricle reduced the respiratory frequency, which was blocked by previous microinjection of bicuculline, a GABA_A_ receptor antagonist (Mayer et al., 2006; Wilson et al., 2004). Furthermore, the expression of A_2A_ receptors was documented in respiratory groups on the ventral surface of the brainstem containing GABAergic neurons, such as the Bötzinger complex, reticular formation, caudal and rostral ventrolateral medula (Zaidi et al., 2006). Zaidi et al. (2006) also described a subpopulation of GABAergic neurons projecting to the phrenic nerve motor nuclei and containing the mRNA for expression of A_2A_ receptors. Further pharmacological studies are required to explore this possibility in control mice, which may contribute to clarifying the mechanisms underlying the increase in respiratory frequency in A_2A_ knockout mice.

It is also important to note that exposure to SH produced an increase in respiratory frequency in Balb/c WT mice, but not in A_2A_KO mice. Indeed, the respiratory frequency of A_2A_KO SH mice was lower in comparison to those maintained under normoxia, suggesting that the adenosine A_2A_ receptors are important for the tachypnoeic response to SH. We may also consider an important role for adenosine and its A_2A_ receptors in the carotid body chemosensory activity in order to explain the absence of increased respiratory rate after SH in A_2A_KO mice. Several studies indicate adenosine as an excitatory mediator of CB hypoxic signaling, and this role is dependent of the activation of A_2A_ and A_2B_ receptor subtypes, which are expressed in CB chemoreceptor cells (Conde et al., 2017; Gauda et al., 2000; Kobayashi et al., 2000; Monteiro & Ribeiro, 1987; Sacramento et al., 2018, 2019; Xu et al., 2006).

Combined with the in vivo recordings of the respiratory parameters, the arterial blood gas analysis provides a full characterization of the respiratory profile in A_2A_ knockout mice and their Balb/c WT controls submitted to SH. In this study we measured arterial pH, $P_{O_{2}}$, $P_{CO_{2}}$ and bicarbonate concentration in order to evaluate possible respiratory disturbances and changes in acid–base balance that may occur in knockout and control mice in response to SH. We observed that after SH both A_2A_ knockout and Balb/c WT control mice presented a decrease in $P_{CO_{2}}$ and an increase in arterial $P_{O_{2}}$. In this case we suggest that peripheral chemoreceptors are activated in response to sustained hypoxia producing a tachypnoeic response in order to increase the oxygen uptake in a situation of low $F_{iO_{2}}$ (Barros et al., 2002; Machado, 2001). It is important to highlight that the measurement of blood gases after SH was performed only after the animals were removed from the hypoxic chamber and the recording of respiratory parameters was completed, that is, several minutes after the animals returned to the normoxic condition. Therefore, the increase in pulmonary ventilation in response to SH for 24 h may explain the increase in $P_{O_{2}}$ and reduction in $F_{iO_{2}}$ in arterial blood of mice when the animals were back to normoxia (21% $F_{iO_{2}}$). Although ventilation did not show a significant increase in A_2A_ knockouts after SH, the observed increase in tidal volume in these animals may impact pulmonary ventilation in order to contribute to the increase in CO_2_ rate elimination and O_2_ uptake.

The reduction in $P_{CO_{2}}$ as a consequence of an increase in the ventilatory response to SH may cause an acid–base imbalance, characterizing respiratory alkalosis. However, no change in arterial pH was observed in A_2A_ knockout and Balb/c WT control mice after SH. The reduced arterial bicarbonate concentration of mice after SH may represent a metabolic compensatory response to the reduction in $P_{CO_{2}}$. We may suggest that an increase in bicarbonate excretion by the kidneys may compensate for the respiratory alkalosis produced by hyperventilation, which may explain the normal pH in mice after SH. Another possibility is that the reduction in blood bicarbonate levels is due to the reduction in $P_{CO_{2}}$, since CO_2_ is transported in the blood mainly in the form of bicarbonate ions. Therefore, further studies are required to confirm that the reduction in bicarbonate concentration in arterial blood of mice after SH represents a compensatory mechanism by the kidneys.

Using a knockout mouse model for A_2A_ receptors combined with cardiovascular and respiratory recordings in conscious freely moving mice, we documented that adenosine A_2A_ receptors play no major role in the modulatory control of the autonomic components to the cardiovascular system as well as in the cardiovascular responses to peripheral chemoreflex activation in mice. On the other hand, the data show a major role for these receptors in the modulation of respiratory frequency because in A_2A_ receptor knockout mice the respiratory frequency was higher than in control WT mice. It is interesting that WT mice submitted to SH presented an increase in the respiratory frequency to levels similar to that observed in A_2A_ KO mice under normoxia, suggesting that in SH the possible inhibitory role played by A_2A_ receptors is removed. In this case, we suggest that SH by a mechanism yet to be revealed is also removing the adenosine inhibitory modulation of the respiratory frequency. Taking into consideration that adenosine is a signalling molecule commonly associated with challenging conditions, such as hypoxia, the increased respiratory frequency in normoxic A_2A_ knockouts actually draws attention to its relevance under physiological conditions, opening interesting possibilities for further studies with the purpose of revealing the mechanisms by which A_2A_ receptors modulate the respiratory frequency.

## AUTHOR CONTRIBUTIONS

J.R.S. and B.H.M. designed the research; J.R.S. performed the experiments; J.R.S. and B.H.M. analysed the data, interpreted results of experiments and wrote the manuscript. Both authors have read and approved the final version of this manuscript and agree to be accountable for all aspects of the work in ensuring that questions related to the accuracy or integrity of any part of the work are appropriately investigated and resolved. All persons designated as authors qualify for authorship, and all those who qualify for authorship are listed.

## CONFLICT OF INTEREST

The authors declare no competing interest.

## Data Availability

The data that support the findings of this study are available from the corresponding author upon reasonable request.
